# Pharmacological Alternatives for the Treatment of Neurodegenerative Disorders: Wasp and Bee Venoms and Their Components as New Neuroactive Tools

**DOI:** 10.3390/toxins7083179

**Published:** 2015-08-18

**Authors:** Juliana Silva, Victoria Monge-Fuentes, Flávia Gomes, Kamila Lopes, Lilian dos Anjos, Gabriel Campos, Claudia Arenas, Andréia Biolchi, Jacqueline Gonçalves, Priscilla Galante, Leandro Campos, Márcia Mortari

**Affiliations:** Neuropharmacology Laboratory, Department of Physiological Sciences, Institute of Biological Sciences, University of Brasília, Brasília 70910-900, Brazil; E-Mails: ju.castroesilva@gmail.com (J.S.); victoriananobio@gmail.com (V.M.-F.); flaviia.medeiros@hotmail.com (F.G.); kamila_farm@yahoo.com.br (K.L.); lilian.dosanjos@gmail.com (L.A.); gabriel_avohay@hotmail.com (G.C.); clauji55@gmail.com (C.A.); andreia.biolchi@gmail.com (A.B.); jacq.coimbra@gmail.com (J.G.); prigalante@yahoo.com.br (P.G.); leandro.ambrosio@gmail.com (L.C.)

**Keywords:** neurological disease, bee venom, wasp venom, polyamine toxins, Melittin, Apamin, AvTx-7, Wasp Kinin, Mastoparan, Pompilidotoxins

## Abstract

Neurodegenerative diseases are relentlessly progressive, severely impacting affected patients, families and society as a whole. Increased life expectancy has made these diseases more common worldwide. Unfortunately, available drugs have insufficient therapeutic effects on many subtypes of these intractable diseases, and adverse effects hamper continued treatment. Wasp and bee venoms and their components are potential means of managing or reducing these effects and provide new alternatives for the control of neurodegenerative diseases. These venoms and their components are well-known and irrefutable sources of neuroprotectors or neuromodulators. In this respect, the present study reviews our current understanding of the mechanisms of action and future prospects regarding the use of new drugs derived from wasp and bee venom in the treatment of major neurodegenerative disorders, including Alzheimer’s Disease, Parkinson’s Disease, Epilepsy, Multiple Sclerosis and Amyotrophic Lateral Sclerosis.

## 1. Introduction

Insect venoms have been used by traditional Chinese and Korean medicine as well as ancient Egyptian and Greek civilizations since 1000–3000 BC to control a number of diseases, including neurological disorders [1,2,3]. Moreover, religious texts such as the Vedas, the Bible and the Koran report the use of bee products to treat diseases [3,4].

The diversity of biologically active molecules from animal venoms is well-known and has long garnered the interest of toxinologists. However, progress is more evident in recent years due to advances in the fields of proteomics, transcriptomics and genomics [5]. The area of venom-based drugs in particular has benefited from these advances along with high throughput screening techniques, which have accelerated the discovery of useful venom-derived drugs.

Bee and wasp venoms are known to be rich in neuroactive molecules that may be valuable in the development of new drugs or act as pharmacological tools to study the normal and pathological functioning of the nervous system [6,7]. As such, this review focuses on the main results obtained for the use of wasp and bee venoms in the treatment of the most prevalent neurodegenerative disorders. It is important to note that several of these compounds could become important new sources for the development of more effective medication with fewer adverse effects. The bioprospection of these compounds is vital since the drugs currently used to treat major neurological disorders (*i.e.*, Epilepsy, Parkinson’s Disease (PD) and Alzheimer’s Disease (AD)) provide only symptomatic relief, and the incidence of serious adverse effects remains high [8,9,10,11].

**Figure 1:**
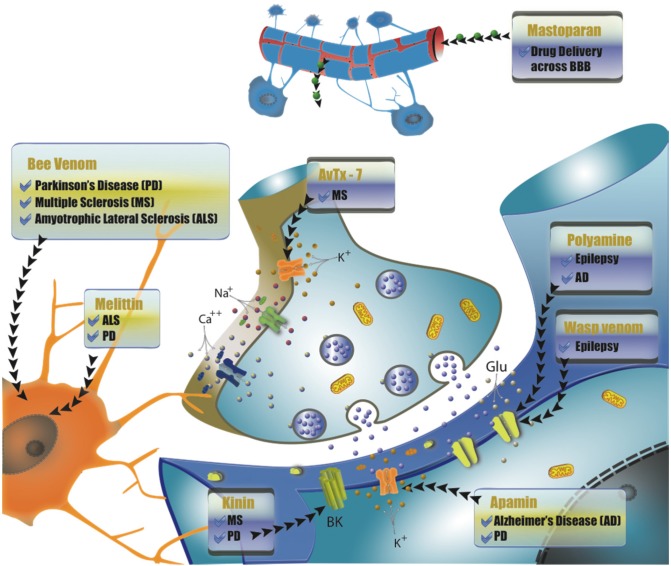
Main targets for wasp and bee venoms in the nervous system according to the type of neurodegenerative disorder treated.

The nervous system is an important target for these toxins, which can modulate synapses as well as generate and propagate action potentials by selectively acting on different ion channels and receptors [12]. Interestingly, evolution has fine-tuned venoms for optimal activity, providing us with a vast array of potential therapeutic drugs, which can be used to design pharmacological agents for the treatment of several diseases, including central nervous system (CNS) disorders [12,13] (Figure 1).

## 2. General Profile of the Main Neurodegenerative Diseases

According to the World Health Organization (WHO), neurological disorders include Epilepsy, Alzheimer’s Disease (AD) and other dementias, Parkinson’s Disease (PD), Multiple Sclerosis (MS), Migraine, Cerebrovascular Disease, Poliomyelitis, Tetanus, Meningitis and Japanese Encephalitis, among others. These diseases are major causes of mortality, accounting for 12% of total deaths worldwide [11]. They are frequently stigmatized, since they are socially incapacitating and can cause cognitive impairment, behavioral disorders, depression and suicide [14,15].

The effectiveness of wasp and bee venom against neurodegenerative diseases has only been investigated for a select group of disorders. Thus, we have performed a brief epidemiological, symptomatic and histopathological summary of the following target diseases: Alzheimer’s Disease, Parkinson’s Disease, Epilepsy, Multiple Sclerosis and Amyotrophic Lateral Sclerosis (ALS).

Among these neurological disorders, neurodegenerative conditions significantly impact not only individuals, but also caregivers and society. The most prevalent neurodegenerative disease is AD, followed by PD and Epilepsy. Neurodegenerative diseases are a heterogeneous group with relentless progression, where aging is a major risk factor in the development [16]. Despite their heterogeneity, all of these diseases are characterized by cognitive impairment, motor alterations and personality changes. Unfortunately, the specific etiology of neuronal death and protein deposition in these diseases remains unknown [16,17].

### 2.1. Alzheimer’s Disease and Other Dementias

Dementia is one of the most frequent causes of cognitive impairment in older adults, with forecasts indicating a worldwide increase from 25 million in 2000 to 115.4 million by 2050. Alzheimer’s alone is responsible for over half of these cases [18,19,20].

Alzheimer’s is symptomatically characterized by memory deficits, cognitive impairments and personality changes [17]. In general, the first clinical signs are impaired short-term memory accompanied by attention and verbal fluency difficulties. Other cognitive functions also deteriorate with the evolution of the disease, including the ability to make calculations, visual-spatial skills and the ability to use everyday objects and tools [17,20].

Estimates indicate the disease will affect more than 80 million people by 2040 and increased life expectancy will see the number of people with AD grow by 300% in developing countries. Since the disease is progressive, patients require prolonged special care after diagnosis, with annual costs estimated at nearly EUR 20,000 per person, exceeding that of patients with cancer [17].

Major contributors to neurodegeneration in brains affected by AD are the deposition of senile plaques, composed primarily of Aβ peptide, and neurofibrillary tangles formed largely by tau protein, which accumulate in neuropils from the cerebral cortex and hippocampus. Moreover, mitochondrial alterations such as fission-fusion abnormalities, defects in electron transport chain proteins, cytoskeletal abnormalities, calcium metabolism, intrinsic apoptosis pathways and caspase activation, as well as free radical generation are also involved in AD pathology [21,22].

More than 100 years after identifying the hallmark lesions in AD, there is still no minimally effective disease modifying therapy available [22]. From 2002 to 2012, of 221 agents submitted to trials for disease-modifying potential, none was different from the placebo in terms of positively affecting primary outcomes [23]. Alzheimer’s treatment is symptomatic and relies on the administration of cholinesterase inhibitors (AChEI) (only tacrine, donepezil, rivastigmine and galantamine are currently approved for AD treatment) and NMDA receptor antagonists (only memantine is approved) [17]. Intervention with AChEI decreases acetylcholine metabolism and enhances neurotransmission, which is associated with memory and cognition reduction in AD [24]. NMDA antagonists act by compensating abnormal tonic activation by glutamate and are more efficient in moderate to severe stages of the disease [25]. Given that these drugs merely provide symptomatic relief, there is an urgent need to develop neuroprotective treatments for AD.

### 2.2. Parkinson’s Disease

Parkinson’s Disease is a universal, incurable, multifactorial and neurodegenerative disorder characterized by gradual degeneration and loss of dopaminergic neurons in the *substantia nigra* (SN). This leads to nigrostriatal pathway denervation, with the presence of Lewy body cytoplasmatic inclusions, predominantly resulting in motor symptomatology. In addition, non-motor symptoms are often identified in PD patients and may precede motor signs [26]. The disorder affects 1% of the population during the fifth or sixth decade of life and is primarily related to aging, with no definitive biomarker available for PD diagnosis [27,28].

Although PD etiology is not yet fully understood, it is possible that a large set of environmental and genetic factors in association with intrinsic neuronal vulnerability in the SN could be involved in the neuronal death typically observed in PD, primarily by inducing oxidative stress and mitochondrial dysfunction [29]. These factors include pesticide exposure, glutamate excitotoxicity, protein misfolding and aggregation, an imbalance in calcium homeostasis and neuroinflammation by microglial activation [30,31]. However, no drug has been clinically proven to modify disease progression, either by protecting surviving dopaminergic cells from degeneration or by restoring lost cells.

In this context, pharmacological treatment for PD remains focused on motor symptoms, mostly by restoring striatal dopamine levels through the administration of dopamine agonists. L-DOPA, a dopamine precursor, is the gold standard for this approach and is often associated with an inhibitor of peripheral degradation (carbidopa and benserazide). Despite its efficiency, long-term L-DOPA treatment is linked to side effects such as motor fluctuations (shorter duration of action) and dyskinesias (abnormal involuntary movements), both of which can significantly reduce quality of life in patients [32,33].

### 2.3. Epilepsy

Epilepsy is an enduring predisposition of the brain to generate epileptic seizures along with the neurobiological, cognitive, psychological and social consequences that the condition causes [34]. More recently it has been defined according to events such as the occurrence of at least two unprovoked (or reflex) seizures in a 24 h period, one unprovoked (or reflex) seizure with the likelihood of further similar seizures, or diagnosis of an epileptic syndrome [35].

Estimates suggest that approximately 65 million people of all ages may be affected by epilepsy [36] and that the majority face treatment problems due to pharmacoresistance to antiepileptic drug (AED) therapy [37,38]. AEDs are classified into three generations, according to their introduction into the market. The first generation of these drugs was sold in the USA and Europe from 1857 to 1958, followed by the second generation between 1960 and 1975. Drugs introduced in the 1960s are potent enzymatic inducers of cytochrome P450 that lead to clinically significant adverse drug interactions and hypersensitive reactions [39]. The 1980s saw the introduction of 15 additional AEDs (third generation), providing more appropriate drug alternatives for patients. However, it is important to underscore that each drug has its advantages and limitations, making treatment a difficult process [40]. Furthermore, these drugs are still inefficient in drug resistant epilepsy, challenging our understanding of the underlying mechanisms of this phenomenon and how to overcome or prevent them. Recent progress in understanding the molecular and cellular events that cause this disease have allowed better management of strategies for the discovery and development of more effective AEDs [41].

### 2.4. Multiple Sclerosis

Multiple sclerosis (MS) is a chronic inflammatory, demyelinating and neurodegenerative disorder of the CNS that begins in young adulthood and may be the result of the interaction between genetic and environmental factors, together with certain pathological hallmarks of an autoimmune disease [42,43,44]. According to the National Multiple Sclerosis Society, the disease affects around 2.1 million people worldwide [45]. MS has a significant socioeconomic impact that is comparable to other neurological conditions. This is because mean disease duration is approximately 38 years, thus affecting individuals at a time when they are entering, developing, or consolidating their professional careers [42].

The pathogenesis of MS is complex and only partially understood, hampering diagnosis and thus the choice of appropriate treatment. Nevertheless, a group of experts recently revised the MS phenotypic classification that includes the five MS subtypes: Relapsing-remitting MS (RRMS), clinically isolated syndrome (CIS), radiologically isolated syndrome (RIS), primary-progressive MS (PPMS) and secondary-progressive MS (SPMS) [46]. Considering the complexity of MS pathophysiology and diagnosis, only a brief description will be given of the main phenotypes included since MS classification began (RRMS, PPMS, and SPMS).

Relapsing-remitting multiple sclerosis (RRMS) represents about 80% of all cases, lasts for about 15 years and is characterized by acute exacerbations from which patients completely or partially recover, with periods of relative clinical stability in between [43,44]. When neurological function declines, the disease progresses to the following stage and is known as primary-progressive multiple sclerosis (PPMS). This type affects 10% of patients, who often present with progressive cerebellar syndrome and myelopathy, or other progressive symptoms [44,47]. Secondary-progressive multiple sclerosis (SPMS) is characterized by a progressive loss of motor function after an initial relapse, occurring about 20 years after the initial event [48]. Furthermore, RRMS is best characterized by an intense focal inflammatory component, whereas PPMS and SPMS exhibit more neurodegenerative features with concomitant chronic inflammation and axon loss [49].

Similar to other neurodegenerative disorders, the limitations of current therapies for MS include lack of superior treatment efficacy, serious adverse effects and long-term safety [43]. Significant advances in the treatment of RRMS are observed when the main goal is to target inflammation and modify the course of the disease; however, the same cannot be said about progressive forms of MS [47,50]. In addition, halting or reversing disease progression is only possible by using remyelinating and neuroprotecting agents, which does not occur in current treatments.

### 2.5. Amyotrophic Lateral Sclerosis

Amyotrophic lateral sclerosis (ALS) is a devastating, progressive and incurable adult-onset neurodegenerative disease characterized by the loss of upper and lower motor neurons in the primary motor cortex, brainstem and spinal cord. The disease affects motor functioning, resulting in paralysis and eventual death, typically from respiratory failure [51,52,53,54,55,56]. Average survival is 3 years after the first symptoms emerge and 5%–10% of patients survive beyond 10 years [57].

The worldwide average incidence rate for ASL is 2.1/100,000 person-years and a point prevalence of 5.4/100,000 persons, strongly linked to increased age [57]. Although little is known about the etiology of ALS, some studies indicate that 10% of cases are familial ALS and 85%–90% are classified as sporadic [53,58,59].

There is increasing evidence that patients with familial and sporadic forms of ALS exhibit signs of multi-modal dysfunction, even in early stages. Previous population-based studies estimated that around 35% of patients exhibit these impairments, including behavioral changes and executive and cognitive function deficits. Furthermore, about 15% of those affected with ALS may also suffer from frontotemporal dementia (ALS-FTD). This leads to reduced quality of life, caregiver stress, clinical effects from ventilator use and gastrostomies, negatively influencing survival time [51,55,60,61,62].

The mechanisms responsible for disease onset and progression remain unknown, hindering the development of targeted therapies for ALS [59]. Given the multifaceted nature of the disease, most of the current approaches employed in clinical trials focus on the emerging concept of stem cell-based therapeutics [59,63]. Riluzole is the only Food and Drug Administration (FDA) approved treatment for ALS and prolongs survival by only a few months [59,64].

## 3. Venoms and Toxins from Wasps and Bees to Combat Neurodegenerative Disorders

The biological capacity to develop a secretion with highly specialized functions and a venomous apparatus is limited to certain groups, including cnidarians, some mollusk families, arthropods, certain reptiles and fish [12]. All insects that can sting are members of the order Hymenoptera, which includes ants, wasps and bees. The most extensively characterized venoms are bee venoms, mainly from the *Apis* genus, as well as some social and solitary wasp genera [4,65].

### 3.1. Bee Venom

Apitherapy is the medicinal therapeutic use of honeybee products, consisting of honey, propolis, royal jelly, pollen, beeswax and, in particular, bee venom (BV). Depending on the disease being treated, BV therapy can be used by applying a cream, liniment, or ointment, via injection, acupuncture or even directly through a live bee sting [4]. However, the most commonly used method is bee venom acupuncture (BVA), which involves the injection of diluted bee venom into acupuncture points. It can be employed as an alternative medicine in patients with PD, pain and other inflammatory diseases, such as rheumatoid arthritis and osteoarthritis [66,67,68].

Bee venom therapy is based on the fact that these crude extracts exhibit a wide variety of pharmacologically active molecules. This pool of chemical compounds is formed by biogenic amine, enzymes (phospholipase A2), basic peptides and proteins (melittin and apamin) and a mixture of water-soluble and nitrogen-containing substances [5].

One of the main biological activities identified in the venom of *Apis mellifera*, the most widely studied honeybee, is the inhibition of inflammatory and nociceptive responses [68]. Studies have shown that inhibition can occur in multiple aspects, making apitherapy the most common application for the treatment of inflammatory diseases such as arthritis, bursitis, tendinitis, rheumatoid arthritis and Lyme Disease [68,69].

Interestingly, BV has also been used in humans to treat neurological diseases with neuroinflammatory aspects, such as multiple sclerosis and Parkinson’s Disease [66,67,70] (Figure 1). Furthermore, several studies on neuroinflammatory diseases in animal models have increasingly supported the effectiveness of this treatment [71,72,73,74] (Table 1).

In regard to anti-neuroinflammatory activity, crude honeybee venom and its components are important tools for the treatment of diseases accompanied by microglial activation [75,76]. Microglia are a population of macrophage cells in the brain that play an important role in immune defense and CNS tissue repair and are vital in controlling normal homeostatic functions in the brain [77].

Under pathogenic conditions, microglia are rapidly overactivated in response to neuronal injury and migrate to the affected sites of the CNS, significantly contributing to neuronal death in specific brain regions [78]. Resting microglia are generally benign to the brain; however, once activated through injury or during removal of unwanted cellular debris, they produce inflammatory cytokines, glutamate, quinolinic acid, superoxide radicals (O_2_^−^) and nitric oxide (NO), undermining cerebral homeostasis.

In this context, the suppression of microglial activation and the neuroprotective effect of BV were observed in several *in vitro* and *in vivo* studies, as well as in clinical trials. Studies in humans have shown that BV may be beneficial in the treatment of diseases that trigger cell death by microglial activation, particularly PD [79,80]. Parkinson’s patients treated with BV acupuncture obtained promising results in idiopathic Parkinson’s Disease Rating Scale Tests [79], demonstrating the remarkable ability of BV acupuncture (BVA) to interfere with PD progression.

**Table 1:** Use of Bee Venom and its components for the treatment of neurodegenerative diseases in *in vivo* models.

Venom or Compound	Neurological Disease	Model Tested	Administration via	Dose	Reference
Bee venom	Parkinson’s Disease	1-methyl-4-phenyl-1,2,4,5-tetrahydropyridine (MPTP) in mice	s.c. acupuncture (point GB34)	0.02 mL bee venom (1:2000 *w*/*v*)	[81]
				once every 3 days for 2 weeks	
Bee venom	Parkinson’s Disease	MPTP in mice	s.c. acupuncture (bilateral point ST36)	A single injection (0.6 mg/kg)	[82]
Bee venom	Parkinson’s Disease	MPTP/probenecid in mice	i.p.	Two injections 3.5 days apart for 5 weeks	[83]
				Low—12 µg/kg/BW	
				High—120 µg/kg/BW	
Bee venom	Parkinson’s Disease	MPTP in mice	i.p.	one i.p. injection	[84]
				BV (1 mg/kg) every day for 6 days	
Bee venom	Parkinson’s Disease	Rotenone-induced oxidative stress and apoptosis	s.c. acupuncture (point GB34)	0.02 mL bee venom (1:2000 *w*/*v*)	[85]
				once every 3 days for 2 weeks	
Bee venom	Multiple Sclerosis	Experimental allergic encephalomyelitis model in rats	-	2 mg/kg or 5 mg/kg	[86]
Bee venom	Amyotrophic Lateral Sclerosis	hSOD1^G93A^ transgenic mice	s.c. acupuncture (bilateral point ST36)	0.1 µg/g—3 times/week for 2 weeks	[87]
Bee venom	Amyotrophic Lateral Sclerosis	hSOD1^G93A^ transgenic mice	s.c. acupuncture (bilateral point ST36) i.p.	0.1 µg/g—3 times/week for 2 weeks	[88]
Apamin	Parkinson’s Disease	MPTP/probenecid mice	i.p.	Two injections 3.5 days apart for 5 weeks	[83]
				Low—0.5 µg/kg/BW	
				High—1.0 µg/kg/BW	
Melittin	Amyotrophic Lateral Sclerosis	hSOD1^G93A^ transgenic mice	s.c. acupuncture (bilateral point ST36)	0.1 µg/g twice a week	[89]

*In vivo* models for BVA and PD have also been tested. Bilateral acupoint stimulation of lower hind limbs prevented the loss of dopaminergic (DA) neurons in the striatum and SN for MPTP-induced PD (1-methyl-4-phenyl-1,2,4,5-tetrahydropyridine) and increased striatal dopamine levels [81,82,83]. MPTP mimics PD in rodents, involving the progressive loss of neurons in SN and causing behavioral alterations typical of PD, making it the most widely used model to study the disease. Chung and colleagues (2012) corroborated the results previously recorded for dopaminergic neuroprotection and observed a reduction in the infiltration of CD4T cells and microglial deactivation in an MPTP-induced PD mouse model [85]. In addition, BVA suppressed neuroinflammatory responses by MAC-1 and iNOS, microglial activation and loss of neurons in SN in the same mouse model [82]. It is important to note that the protective effect of BV on DA neurons of the SN is not restricted to acupoint stimulation, since it is also observed when using intraperitoneal injections [83].

Recently, an extensive and important study indicated that BV was capable of normalizing neuroinflammatory and apoptotic markers and restoring brain neurochemistry after simulated PD injury in mice [85], revealing the significant potential of BV application for PD therapy. Moreover, BV exhibited no signs of toxicity on general physiological functions when administered subcutaneously within a higher therapeutic range (100–200 fold) [90].

In *in vitro* tests, BV reduced the production of NO, COX-2, PGE2 and pro-inflammatory cytokines (IL-1β, IL-6 and TNF-α) in murine microglia cultures stimulated by lipopolysaccharides (BV-2 cell line) [75,91,92,93,94]. Additionally, tests using SH-SY5Y human neuroblastoma cells and MPTP demonstrated an increase in cell viability, reduced apoptosis by DNA fragmentation assays, and inhibited cell death cascade activation after pre-treatment with BV [95].

Bee venom has also been investigated in the treatment of MS and ALS (Table 1). In 2007 and 2008, two reviews summarized relevant findings regarding the therapeutic potential of venoms and other non-conventional approaches in MS treatment [80,96]. An interesting cross-sectional study involving 154 patients with MS investigated how often they used complementary and alternative medicine (CAM), including apitherapy [97]. The authors concluded that about 61% used CAM, and more than 90% of these used it as an adjunct to allopathic treatments. Furthermore, 65.8% of the interviewees reported an improvement. Given its importance and the growing interest in BV therapy, the American Apitherapy Society began to track patients who receive this treatment regularly, enrolling over 6000 members who take BV for MS or rheumatoid arthritis [98].

An FDA—approved investigational new drug trial involving nine patients with progressive MS evaluated the safety of BV [70]. Intradermal injections of gradually increasing doses were administered for 17 weeks until treatment reached 2.0 mg/week. A questionnaire, functional neurological tests and changes in measurement of somatosensory-evoked potentials were used to assess responses to therapy. None of the subjects displayed severe allergic reactions, although four reported worsening neurological symptoms and had to discontinue treatment. Two other patients showed objective improvement and three exhibited subjective symptom improvement. This was a preliminary study performed on a small number of patients and, despite the few positive results obtained, it was difficult to establish definitive conclusions regarding the efficacy of apitherapy.

In the same year, a high quality clinical trial for apitherapy in MS [99] evaluated the effectiveness of BV in 26 relapsing-remitting or secondary progressive MS patients [100]. This crossover study tested two groups; one received bee sting therapy for 24 weeks and placebo for another 24 weeks, while the other was given the same treatments in reverse order. Live bees were used to administer BV three times a week, with an increasing number of stings in each session to a maximum of 20 bee stings. Although it was well tolerated with no serious adverse events, the therapy failed to reduce fatigue, disease activity or disability, or improve quality of life. By contrast, phase II of the study assessed the efficacy of BV in patients with either RRMS or chronic progressive MS and found that BV intradermal injections decreased functional debilitation [101]. Treatment was administered until positive clinical effects reached a plateau, with an initial dose of one bee sting. In general, more than 68% of patients experienced some beneficial effects from BV therapy, including better balance, coordination, bladder and bowel control, as well as improved extremity strength, fatigue, endurance, spasticity and numbness, providing important evidence for the use of BV in MS. The authors attributed most of the positive findings to patients suffering from chronic-progressive MS when compared to relapsing-remitting MS, largely due to inherent variability among these MS patients, hindering result assessment.

A more recent study showed significant positive effects attributed to BV treatment in an experimental allergic encephalomyelitis animal model for MS induced by guinea pig spinal cord homogenate [86]. The results indicate that BV significantly decreases clinical symptoms and immunization effects in Lewis rats, as well as penetration of inflammatory cells and serum TNF-α and nitrate levels.

Considering all the findings reported on BV therapy for MS and according to Namaka and collaborators (2008), the different results reported to date may be due to the therapeutic protocols used, type of animal model and/or type of challenged cell line, in addition to potential time and dose-dependent properties [96].

Bee venom has also been studied for the treatment of ALS. A study using a symptomatic animal model for ALS with mutant hSOD1^G93A^ transgenic mice showed an improvement in motor activity in the rotarod test and prolonged life span for mice treated with BV acupoint stimulation [87]. The results obtained were substantiated by reduced levels of cytokines, typically released by activated microglia and astrocytes, leading to the neuroprotective effect observed. Moreover, by contributing to the reduction of motor neuron degeneration, BV prevented mitochondrial disruption and activated cell survival signal transduction pathways.

Research using the same animal model found that transgenic mice that received BV exhibited reduced expression of α-synuclein modifications, ubiquitinated α-synuclein and recovered spinal cord proteasomal activity [102]. It is important to underscore that animals received only two subcutaneous injections of 0.1 μg/g of BV at an acupoint, which was sufficient to induce positive effects.

Interestingly, another recently published study compared the effects of BV treatment using different administration routes for the same symptomatic model of ALS [88]. It was noted that BV treatment through an acupoint was more effective than intraperitoneal (i.p.) BV administration and acupoint stimulation alone. The results demonstrated an improvement in walking function, lower levels of neuroinflammatory proteins (TLR4, CD14 and TNF-α) in the spinal cord and reduced nuclear abnormality in the quadriceps femoris muscle.

In a study evaluating the ability of BV to act on the impaired ubiquitin-proteasome system [103], NSC34 motor neuronal cells expressing the mutant gene hSOD1^G85R^ were used and stimulated with 2.5 μg/mL of BV for 24 h. Once again the results showed restored proteasome activity and a reduction in the amount of misfolded SOD1. However, BV did not activate the autophagic pathway in these cells, a process frequently impaired in ALS that results in the aberrant accumulation of misfolded and/or aggregated proteins within spinal cord cells. This BV effect is remarkable because it reduces protein aggregation by targeting the ubiquitin system as opposed to activating the autophagy pathway.

Thus, when taken together, these findings reinforce the therapeutic potential of BV treatment, demonstrating an antineuroinflammatory effect, reduced neuronal loss caused by misfolded protein aggregates and glutamate neurotoxicity, restoration of the ubiquitin-proteasome system and motor improvement. These results could have important clinical implications for BV use as a coadjuvant treatment in both ALS and other neurodegenerative disorders.

### 3.2. Wasp Venom

With respect to wasps, important studies reveal the pharmacological potential of these venoms, present primarily in the *Polybia* genus (Table 2). In 2005, Cunha and colleagues described the effects on rats of an intracerebroventricular (i.c.v.) injection of crude and denatured venom of the social wasp *Polybia ignobilis* [104]. Interestingly, crude venom provoked severe generalized tonic-clonic seizures, respiratory depression and death. On the other hand, denatured venom had an antiepileptic effect on acute seizures induced by i.c.v. injection of bicuculline, picrotoxin and kainic acid, but not on pentylenetetrazole(PTZ)-induced seizures. In addition, the denatured venom inhibited [^3^H]-glutamate binding in membranes from the rat cerebral cortex at lower concentrations than those used for [^3^H]-GABA binding [105]. These results indicate that specific components in the venom of *P. ignobilis* may interact with GABA and glutamate receptors, representing a significant source of neuroactive molecules (Figure 1).

**Table 2:** Use of Wasp Venom and its components for the treatment of neurodegenerative diseases in *in vivo* models.

Venom or Compound	Neurological Disease	Model Tested	Route of Administration	Dose	Reference
Denatured venom—*P. ignobilis*	Epilepsy	Acute seizures model induced by chemoconvulsants in rats	i.c.v.	400 μg/animal	[104]
Denatured venom—*P. occidentalis*	Epilepsy	Acute seizures model induced by chemoconvulsants in rats	i.c.v.	120, 240 and 300 μg/animal	[105]
Low molecular weight compounds—*P. occidentalis*	Epilepsy	Acute seizures model induced by PTZ	i.c.v.	70, 210 and 350 μg/animal	[106]
Bradykinin	Stroke	Transient forebrain ischemia in rats	i.p.	150 μg/kg 48 h after ischemia	[107]
Bradykinin	Stroke	Transient forebrain ischemia in rats	i.p.	150 μg/kg 48 h after ischemia	[108]

Similarly, i.c.v. administration of the denatured venom of *P. occidentalis* inhibited epileptic seizures caused by the same chemical convulsants previously described and was ineffective against PTZ-induced seizures [105]. A subsequent study with low molecular weight compounds (LMWC) from *P. paulista* wasps demonstrated their ability to block PTZ-induced seizures [106]. This effect is likely due to the presence of different compounds that act on GABA receptors.

Finally, research on crude venom from the social wasp *Agelaia vicina* revealed its ability to competitively inhibit high- and low-affinity GABA and glutamate uptake [109]. This is an important result since diseases such as Stroke, Epilepsy and PD involve abnormalities in GABA and glutamate uptake systems [110,111].

## 4. Compounds Isolated from Wasp and Bee Venom for the Treatment of Neurodegenerative Diseases

In addition to crude venom, several venom components have been widely used in Oriental medicine to relieve pain and treat inflammatory diseases such as rheumatoid arthritis and tendinitis [68,69,112]. Other potential venom-related treatments include the inhibition of neuroinflammatory responses, useful in the treatment of PD, AD and MS. This section of the review highlights the most recent and innovative therapeutic and biological applications of bee venom compounds: Melittin and Apamin (Table 1); and wasp venom compounds: Pompilidotoxins, Mastoparans, Kinins and Polyamine toxins (Table 2).

### 4.1. Peptides from Bee Venom as Therapeutic Sources

#### 4.1.1. Melittin

**Figure 2:**
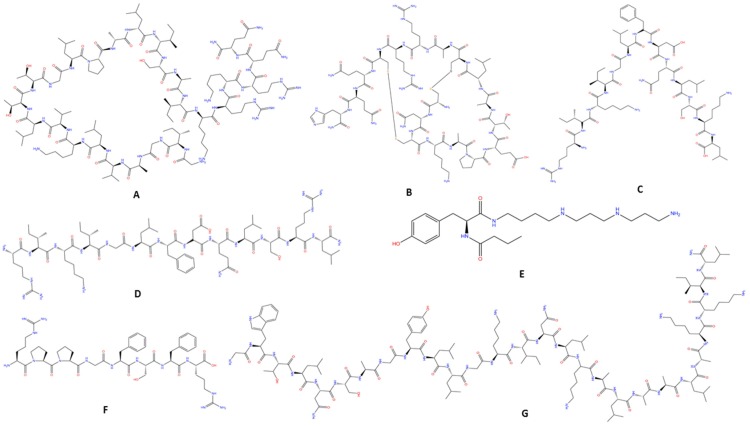
Chemical structures of compounds found in bee and wasp venoms. (**A**) Melittin [119]; (**B**) Apamin [120]; (**C**) Alpha-pompilidotoxin [121]; (**D**) Beta-pompilidotoxin [122]; (**E**) Philanthotoxin [123]; (**F**) Bradykinin [124]; (**G**) Transportan [125].

Melittin is the main component found in BV, accounting for 40% to 60% of dry venom, and is the best characterized peptide in BV. This linear peptide has 26 amino acid residues, alkaline characteristics, a predominantly hydrophobic *N*-terminal region and a hydrophilic *C*-terminal, resulting in amphiphilic properties [113] (Figure 2A). It appears to be primarily responsible for intense local pain, inflammation, itching and irritation in higher doses. On the other hand, in very small doses Melittin can cause a wide range of central and systemic effects, including anti-inflammatory effects, increased capillary permeability and lower blood pressure, among others [114].

The effect of Melittin on the CNS has been documented since 1973, when studies showed its marked effect on inhibiting general behavior, exploratory activity and “emotionality”, in addition to disrupting spontaneous and evoked bioelectric activity in the brain. Moreover, high doses of this peptide can induce a depressant effect evaluated by electroencephalography in anesthetized cats. This effect was associated with reduced systemic blood pressure [115,116].

In 2011, Yang and collaborators studied the therapeutic effect of Melittin in a transgenic mouse model for ALS. In this model, Melittin-treated animals exhibited a decline in the number of activated microglia and expression of proinflammatory factor TNF-α, inhibiting the increased neuroinflammation responsible for neuronal death in this disease. Moreover, Melittin regulates the production of misfolded proteins by activating chaperones and alleviating α-synuclein post-translational modification, an important mechanism for PD and ALS pathologies. Melittin also restored proteasome activity in the brainstem and spinal cord. Interestingly, treatment with this alkaline peptide in a symptomatic ALS animal model improved motor function and reduced neuronal death [117].

Additionally, *in vitro* assays revealed the potential in Melittin as an agent for the prevention of neurodegenerative diseases, considering its ability to inhibit the apoptotic factor and cell death in neuroblastoma SH-SY5Y cells [118]. Melittin also demonstrated a potent suppressing effect on proinflammatory responses for BV2 microglia by reducing proinflammatory mediators and production of NO, PGE2 and cytokines [89]. Thus, it is suggested that this compound may have significant therapeutic potential for the treatment of neurodegenerative diseases accompanied by microglial activation, such as PD (Figure 1).

Recently, Dantas and colleagues (2014) investigated the pharmacological effects of Melittin on the nervous system of mice [126]. The animals were submitted to behavioral tests, including the catalepsy test, open field and apomorphine rotation tests. The results showed that mice treated with Melittin displayed no cataleptic effects or changes in motor activity, although there was a reduction in the effects induced by the apomorphine test. As such, the authors found that Melittin exhibited antipsychotic properties and may be an alternative for the treatment of psychotic diseases, reducing the classic side effects caused by conventional neuroleptic drugs.

#### 4.1.2. Apamin

Neurotoxin Apamin is the smallest peptide, accounting for less than 2% of BV, with 18 amino acids residues, a high cysteine content and alkalinity (Figure 2B). Moreover, it is well known for its pharmacological property of irreversibly blocking Ca^+^ activated K^+^ channels (SK channels) and is considered the most widely used blocker for this type of channel [113,127].

Small-conductance Ca^2+^-activated K^+^ (SK) channels control the firing frequency of neurons, especially at AMPA and NMDA glutamatergic synapses, and are responsible for hyperpolarization following action potentials [128]. These channels can be positively or negatively modulated. Positive modulation involves binding the compound, which then facilitates channel activity, thus impairing memory and learning. The opposite is true for negative modulation, where memory and learning improve and calcium channel sensibility declines [129]. Apamin acts through the second mechanism described. In neurons, this SK channel blockage decreases hyperpolarizing effects, modulating synaptic plasticity and memory encoding [130,131]. In addition, when compared to other arthropod neurotoxins, Apamin has an unusual ability to cross the blood brain barrier (BBB) and acts mainly in the CNS, where SK channels are extensively expressed [130].

Alvarez-Fischer *et al.* (2013) studied the protective effect of this peptide on dopaminergic neurons in a chronic mouse model of MPTP-induced PD [83]. The animals received i.p. injections in two different dosages of Apamin (low: 0.5 µg/kg; high: 1.0 µg/kg) in order to assess brain lesions and behavioral effects in mice. Results showed that Apamin protected nigral DA neurons and increased striatal DA levels in the nerve terminals. In the behavioral test, data were paradoxical, indicating that mice treated with Apamin spent significantly less time on the spindle in comparison to saline-treated animals with MPTP brain lesions, despite the authors’ suggestion that Apamin may improve neuroprotection of dopaminergic neurons [83]. In this context, cell cultures that mimic the selective demise of mesenphalic dopaminergic neurons showed a lower degeneration rate after Apamin treatment [132]. Furthermore, Apamin has also been evaluated for the treatment of PD using the motor score from the Unified Parkinson’s Disease Rating Scale. In this study, Apamin exhibited primarily neurorestorative activity in PD, as well as symptomatic and neuroprotective activity [133].

Several behavioral and electrophysiological studies have suggested Apamin in the treatment of AD, indicating that the blockage of SK channels by this compound may enhance neuronal excitability, synaptic plasticity, and long-term potentiation in the CA1 hippocampal region (Figure 1) [134]. Likewise, Apamin is a valuable tool in the investigation of physiological mechanisms involved in higher brain functions, such as cognitive processes or mood control, and there is already a patented method for early diagnosis of AD using Apamin [135,136,137,138,139]. However, it is important to underscore that SK blockage may accelerate neurodegenerative processes, making additional research in this field imperative.

### 4.2. Wasp Venom Peptides as Therapeutic Sources

#### 4.2.1. Pompilidotoxins

Pompilidotoxins are a group of neuroactive molecules that were first described by Konno *et al.* [140,141]. They consist of two neurotoxins known as α- and β-pompilidotoxin (PMTX), derived from solitary wasps *Anoplius samariensis* and *Batozonelus maculifrons*, respectively. These molecules are peptides composed of 13 amino acid residues, differing solely in the presence of an amino acid at position 12, corresponding to lysine in α-PMTX and Arginine in β-PMTX (Figure 2C,D, respectively). This minimal structural difference appears to be responsible for the significant potency of β-PMTX, approximately five times higher than α-PMTX, when tested in the lobster neuromuscular junction. Moreover, both peptides act on mammalian central neurons, primarily by blocking Na^+^ current inactivation [142].

It has been demonstrated that α-PMTX interrupts synchronous firing of rat cortical neurons, facilitates synaptic transmission in hippocampal slices and decelerates the inactivation of tetrodotoxin-sensitive voltage-gated sodium channels (VGSCs) from rat trigeminal neurons [143,144]. In turn, β-PMTX modulated spontaneous rhythmic activity in spinal networks [145] and acted on hippocampal CA1 neurons by interfering with postsynaptic potential, increasing excitatory potential and interrupting rapid inhibitory potential [146]. Given that the main action of Pompilidotoxins is to slow the inactivation of VGSCs, these peptides may provide a better understanding of the molecular determinants associated with alterations in these channels involved in neuropathological conditions. The alteration of sodium channels has been described as a contributor to the events involved in several neurological disorders, especially persistent sodium currents that can participate in the physiopathology of some types of epilepsy and MS [147,148]. It is important to note that finely orchestrated activation and inactivation is essential for the correct maintenance of neuronal excitability and the slightest change in this equilibrium can result in serious consequences for the individual.

#### 4.2.2. AvTx-7

Research by Pizzo *et al.* (2004) showed that neuroactive peptide Avtx7, isolated from the venom of social wasp *Agelaia vicina*, acted on the blockage of tetraethylammonium and 4-aminopyridine (4-AP)-sensitive K^+^ channels (Figure 1) [149]. As such, this novel neurotoxin may be a valuable tool in better understanding how K^+^ channels work on neurological diseases, such as dementia and MS. These results were obtained using cortical brain synaptosomes and by assessing glutamate release as a response to different potassium blockers. K^+^ channels are critically involved in the nervous system, consequently, alterations in their function can lead to important perturbations in membrane excitability and neuronal function. For instance, the dysfunction of a subfamily or subtype of K^+^ channels might induce AD or PD [150]. Thus, K^+^ channel blockade, for instance by 4-AP, has been linked to an action potential extension with a consequent increase in duration, which is relevant for the treatment of MS. Since 1990, the use of 4-AP in patients with MS has been described to reduce fatigue and improve visual field defects [151]. However, despite its therapeutic effects, drawbacks include low selectivity, causing severe adverse effects and difficulty determining individual therapeutic dose. In this respect, research targets more selective pharmaceuticals to treat MS by using these blockers, though with fewer side effects [152].

In regard to potassium blockers, an important line of research proposes their use as a meaningful non-dopaminergic alternative for the treatment of neurodegenerative diseases, such as advanced-stage PD. The use of these blockers is favorable in three mechanisms: Increased neurotransmitter release (*i.e.*, glutamate), modulation of neuronal network oscillation and greater cortical excitation. In relation to 4-AP, advanced clinical trials have shown satisfactory results, leading to FDA approval in 2013 for the treatment of movement dysfunction in patients with MS [153]. In this field, the discovery and identification of AvTx7 provides new pharmacological options, since its mechanism seems to be related to 4-AP.

#### 4.2.3. Mastoparan

Mastoparan is a class of multifunctional peptides found in solitary and social wasp venom, with its primary activity described in mast cell degranulation, giving the peptide its name [154]. Thus, these peptides exhibit a number of remarkable pharmacological activities, such as antimicrobial, antitumor, insulinotropic and neurological effects [114,155,156,157,158,159].

The first Mastoparan was identified and chemically characterized by Hirai *et al.* in 1979, when this molecule was isolated from the social wasp *Vespula lewisii*. Mastoparans are short cationic peptides with 10 to 14 amino acid residues, two to four lysine residues and *C*-terminal amidation, characteristics that are essential for proper peptide action [160,161]. These peptides can interact and penetrate biological membranes via the positively charged side-chains of their amphipathic α-helical structures [161]. In light of this property, Mastoparans were recently classified as cell-penetrating peptides (CPP) [162].

Crossing the BBB is a significant challenge in neuropharmacology. The BBB is responsible for regulating brain homeostasis through selective permeability that protects the CNS. However, these characteristics also affect drug delivery and bioavailability to the CNS. Advances in the fields of pharmacokinetics, molecular biology, nanotechnology and toxinology have resulted in strategies to facilitate the crossing of drugs through the BBB, thus, increasing drug concentration in the brain [163]. Cell permeable peptides (CPP), particularly Mastoparans, serve as vehicles for the delivery of different molecules and particles into the brain and neurons and have been studied in combination with compounds that act on the CNS [164].

With the aim of enabling neuroactive compounds to permeate the BBB, researchers have created new chimeric peptides (Transportan), connecting Mastoparans and the neuropeptide Galanin in two different ways. The first compound, named Transportan, is formed by 12 residues of Galanin and a full length Mastoparan connected by a lysine, resulting in a chimera with 27 residues [164] (Figure 2G). The second compound, called Transportan 10, consists of seven terminal residues of Galanin and a full Mastoparan connected by a lysine residue [165].

Galanin, discovered in 1983, is a neuropeptide that in humans contains 30 amino acid residues and 29 in other species, for revision see [166]. Its name originates from the fusion of Glycin and Alanin, the *N*-terminal and *C*-terminal amino acids, respectively. Widely distributed in the peripheral and central nervous systems, Galanin has been associated with the pathophysiology of neurodegenerative diseases such as AD and Epilepsy [166]. Several studies report that the overexpression of Galanin detected in AD can preserve cholinergic striatal neuron function, which in turn may slow AD symptoms [167]. The chimeric construction of Transportan and Transportan 10 has been used as a drug delivery system for Galanin in the CNS and as treatment for neurodegenerative diseases, acting as a neuroprotective agent (Figure 1).

Another important function of Mastoparans is that they act as an antidote to one of the most powerful neurotoxins in the world, Botulinum toxin A (BoTx-A). If inhaled, only one gram of crystallized BoTx-A dispersed in the air can kill a million people [168]. Intoxication is so rapid and severe that some countries developed biological weapons containing BoTx for use in World War II. Intoxicated patients are treated with serum therapy. However, this does not reverse the toxic effects already induced in the organism [169]. As such, in an effort to treat this intoxication, a group of researchers employed Mastoparan 7 as a CPP in a chimeric construction denominated Drug Delivery Vehicle-Mas 7 (DDV-Mas 7). Consisting of a non-toxic heavy chain fragment of BoTx-A and Mastoparan 7, this chimeric peptide induced neurotransmitter release in a culture of mice spinal cord neurons, reversing the effect of the BoTx-A and allowing Ach liberation, followed by muscular contraction [160].

Mastoparans also modulate G-protein activity without receptor interaction, currently considered a preeminent tool for the study and understanding of this complex intracellular signaling system [170,171,172,173]. Several neurological disorders, including Mood Disorders, Epilepsy, AD, and PD are related to G protein-coupled receptors [174,175,176]. Thus, over the last decade, natural, modified or chimeric Mastoparans have been used as a potential treatment for a number of neurological conditions.

#### 4.2.4. Wasp Kinin

Another class of peptide frequently encountered in wasp venom is Kinin, composed of Bradykinin (BK) and its analogues, largely responsible for the pain caused after a wasp sting and the paralyzing action used for prey capture [177,178,179]. Naturally present in different animals, BK was first described in 1949 by Rocha and Silva as consisting of nine amino acid residues (Figure 2F), with its primary activity described in mammal platelets [180]. This small peptide plays an important role in controlling blood pressure, renal and cardiac function, and inflammation [181]. It is important to note that Kinin was the first neurotoxin component isolated from wasp venom. In addition, Kinin acts on the insect CNS, where it irreversibly blocks the synaptic transmission of nicotinic acetylcholine receptors [179,180,181,182]. Furthermore, Kinin components, produced via the kallikrein-kinin system, have been found in abundance throughout both the rat and human CNS attracting interest in neuroprotective research [183] (Figure 1). Two major Kinin receptor families have been identified: B2 and B1 receptors. Their expression is low under normal conditions, but is up-regulated following injury, infection and inflammation [184].

Although several studies report that BK likely triggers a specific cascade of inflammatory events in the CNS, it has also been shown to possess anti-inflammatory (neuroprotective) properties, suppressing the release of inflammatory cytokines (TNF-α and IL-1β) from microglia in *in vitro* assays [183]. According to these authors, BK modulated microglial function by negative feedback for cytokine production, increasing prostaglandin synthesis and causing greater microglial cAMP production [183].

BK can also be beneficial after ischemic stroke, particularly if administered in the latter stages as opposed to the initial phases, where its harmful effects include inflammatory response and neurogenic inflammation [185]. It is noteworthy that molecular and functional evidence has suggested that interaction with B1 receptors may provide a new therapeutic approach in MS, primarily by reducing the infiltration of immune cells (lymphocytes T) into the brain [184]. Additionally, treatment with BK applied two days after transient forebrain ischemia in rats in post-conditioning studies provided 97% neuroprotection for the particularly vulnerable CA1 hippocampal neurons, as well as a decrease in Caspase3 expression and iNOS-positive cells, and also a suppression in the release of cytosolic cytochrome *c* and MnSOD [107,108]. This indicates that the neuroprotective mechanism initiated by BK may also inhibit the mitochondria-mediated apoptotic pathway [108]. The neuroprotective role of BK has also been reinforced by evidence of its action in the retina, protecting against neuronal loss induced by glutamatergic toxicity. This BK-induced protection caused a downstream reaction in NO generation and an upstream reaction in radical oxygen generation [186].

As observed, BK agonists may provide a new platform for drugs designed to treat neurodegenerative disorders that involve microglial activation, such as PD and acute brain damage. In this respect, wasp venom contains a multitude of Kinins with different activity potency profiles. A good example is Thr^6^-Bradykinin, a compound isolated from several wasp venom samples. The single substitution of serine for threonine in this compound results in enhanced action when compared to BK. According to Mortari *et al.* (2007), this peptide displays remarkable anti-nociceptive effects when injected directly into the rat CNS; it is approximately three times more potent and remains active longer than BK [187]. These results can be explained by a more stable conformation in its secondary structure and/or the modification may protect against hydrolysis through neuronal kininases, preserving the effect of the peptide on B2 receptors [187,188].

### 4.3. Polyamine Toxins as Therapeutic Sources

Polyamine toxins are a group of low molecular weight (<1 kDa), non-oligomeric compounds isolated primarily from the venom of wasps, followed by spider venoms [189,190] (Figure 2E). The first polyamine toxin described, Philanthotoxin-433 (PhTX-433), was isolated from the venom of the wasp *Philanthus triangulum* [191]. These small natural molecules exhibit a number of biological activities and have been used as tools in the study of ionotropic glutamate (iGLU; AMPA) and nicotinic acetylcholine (nACh) receptors since the 1980s [190,192,193]. Interest is centered on its action as a non-selective and potent antagonist of glutamate receptors in the invertebrate and vertebrate nervous system (Figure 1) [192,193,194]. Moreover, it is believed that the abnormal activation of iGLU receptors is involved in neurological and psychiatric diseases such as AD, PD, Stroke, Depression, Epilepsy, Neuropathic Pain and Schizophrenia [195,196].

With respect to iGLU, current polyamine toxins (PhTXs) and their derivatives have the ability to differentiate which AMPA receptors are in fact permeable to Ca^2+^ ion, acting as a non-selective open-channel blocker [190,197]. As a result, PhTXs can control the excessive opening of overactivated ion channels (due to pathological conditions) and block the exaggerated influx of calcium, culminating in neuroprotection [193,198]. Interestingly, this mechanism of action is similar to that of Memantine, a drug used in the symptomatic treatment of moderate to severe AD [199]. Thus, the existence of a drug that has obtained good clinical results and its similarity with polyamine toxins illustrates the potentially promising role of these molecules and highlights the need for further research.

Recently, a computational model approach was devised to better understand how polyamine toxins interact with ion channels coupled with glutamate receptors [200]. This study found that these molecules could bind to the narrowest central region of the ion channel and block local ion flow. Membrane potential is important in toxin-receptor interaction, and as such, polyamine toxins are generally highly voltage-dependent blockers of iGLU [200]. In this regard, Nørager *et al.* recently developed fluorescent templates using polyamine toxin analogues to visualize these ligands in iGLU of living tissue [201].

## 5. Conclusions

Due to the rising prevalence of neurodegenerative diseases among the elderly, there is a pressing need for better treatment to alleviate the social and financial burden of these disorders. There are multiple targets for treating neurodegenerative diseases, considered complex syndromes that are difficult to control in a stable and lasting manner. Effective treatment of these diseases may require that the different pathogenic events associated with neurodegenerative diseases, such as the clearance of disaggregated proteins targeted in conjunction with neuroprotective and immunomodulatory strategies. In this respect, therapy using bee and wasp venoms is considered a psychoneurological approach for autoimmune and neurodegenerative diseases. Since these venoms contain a number of compounds, mainly peptides, advances in modern identification and sequencing techniques have facilitated and subsidized the elucidation of their full composition, thus providing an arsenal of new possibilities to combat a series of neurodegenerative diseases, using different neuroactive mechanisms of action.
